# Identification of a SARS-CoV-2 virus-encoded small non-coding RNA in association with the neurological disorders in COVID-19 patients

**DOI:** 10.1038/s41392-022-00969-1

**Published:** 2022-03-31

**Authors:** Qian Zhao, Qiong Wang, Bing Zhao, Yixing Wang, Jinhui Lü, Yuefan Guo, Xiaoping Zhu, Lu Qian, Shanshan Yu, Lipeng Hao, Zhongmin Liu, Zuoren Yu

**Affiliations:** 1 Key Laboratory of Arrhythmias of the Ministry of Education, Research Center for Translational Medicine, Heart Failure Institute, Shanghai East Hospital, Tongji University School of Medicine, Shanghai, China; 2 Shanghai Pudong Center for Disease Control & Prevention, Pudong New Area, Shanghai, China; 3 Department of Internal Medicine of Traditional Chinese Medicine, Tongji University School of Medicine, Shanghai East Hospital, Shanghai, China

**Keywords:** Prognostic markers, Non-coding RNAs

**Dear Editor**,

Coronavirus disease 2019 (covid-19), caused by severe acute respiratory syndrome coronavirus 2 (SARS-CoV-2), has led to 460 million cases confirmed and 6.1 million deaths across almost 200 countries until March 2022. In addition to the vaccine-based protection against viral infection, it is urgently needed to accurately diagnose covid-19 patients as early as possible after infection. The development of a reliable diagnostic method with high sensitivity and high accuracy will be of great significance to determine whether SARS-CoV-2 infection occurs in close contacts during the incubation period before showing any symptoms. This will lead to accurate diagnosis and timely quarantine at the very beginning of the viral infection, which will not only minimize the spread of the virus but also refrain patients from severe symptoms by preventive medical treatment immediately after early diagnosis.

In the last two decades, small non-coding RNAs including microRNA (miRNAs) have attracted the attention of scientists in the field of life science due to their regulatory function of gene expression and diagnostic potential for human diseases. miRNAs have been well studied in mammals. Literature has reported viruses-encode miRNAs, named vmiRNAs.^1^ So far, nearly 500 precursors or mature vmiRNAs have been successfully identified and registered in the miRNA database (miRBase.org). RNA viruses-encoded vmiRNAs have been identified in HIV retrovirus, Ebola filovirus, as well as SARS coronavirus.^2–4^ More importantly, a vmiRNA identified from the serum of Ebola-infected patients was found to be detectable earlier than the viral genome, most likely due to secretion of exosomal vmiRNA prior to circulating viruses in patients.^2^ VmiRNAs encoded by SARS-CoV and SARS-CoV-2 were predicted by Khan et al. utilizing computational approaches.^3^ Broad target interactions between the SARS viral miRNAs and immune-signaling pathways were indicated.^3^ So far, MR147-3p is the only vmiRNA primarily predicted from the SARS-CoV-2 viral genome, and further validated in the SARS-CoV-2-infected Vero E6 cells.^4^ However, detection of vmiRNAs in SARS-CoV-2-infected patients has not been reported yet.

Herein, through computational prediction of the SARS-CoV-2 viral genome sequence and vmiRNA screening analysis of the samples from covid-19 patients, we successfully identified a SARS-CoV-2-encoded vmiRNA and its precursor (pre-vmiRNA) with a hairpin secondary structure in the region of ORF1a (Fig. 1a). It was named pre-CvmiR-5 to the precursor, and CvmiR-5-5p to the mature vmiRNA. A sequence blast analysis with nine kinds of coronavirus indicated that the CvmiR-5-5p sequence was specific to SARS-CoV-2, and conserved among SARS-CoV-2 and its two main mutants including Delta strain and Omicron strain (Supplementary Fig. S1). To validate the biogenesis of CvmiR-5-5p in human cells after viral infection, pre-CvmiR-5 was synthesized and cloned into pcDNA3.1 plasmid (Supplementary Fig. S2), followed by transfection into human alveolar basal epithelial cell line A549. Quantitative RT-PCR analysis demonstrated the biogenesis of CvmiR-5-5p in a dose-dependent manner, while undetectable in control cells (Supplementary Fig. S3a and Fig. 1b). Exosomal CvmiR-5-5p in the supernatant at different time points showed a similar pattern with the intracellular CvmiR-5-5p (Supplementary Fig. S3b). Agarose gel electrophoresis of the PCR products indicated a similar size between mature CvmiR-5-5p and internal control hsa-miR-16-5p (Supplementary Fig. S4). The PCR products were further applied for sequencing. The sanger sequencing diagrams derived from 37 clones directly demonstrated the biogenesis of mature CvmiR-5-5p in the host cells with diverse isomiRs between 20–23 nt in length (Fig. 1c). In addition, a pseudovirus fragment starting from 5′ end to nt 2400 of SARS-CoV-2 covering pre-CvmiR-5 was transfected into A549 cells, leading to the biogenesis of mature CvmiR-5-5p in a dose-dependent manner (Supplementary Fig. S5). In order to further validate the biogenesis of CvmiR-5-5p from the whole genome of SARS-CoV-2 in the infected cells, a dataset (GSE148729)^5^ was derived from a small RNA sequencing analysis of SARS-CoV-2 virus-infected Calu-3 cells showed 419 reads matching CvmiR-5-5p and its precursor. The sequence alignment demonstrated the enrichment of CvmiR-5-5p with ~20–23 nt in length (Fig. 1d), which was consistent with the results in Fig. 1c.

**Fig. 1:**
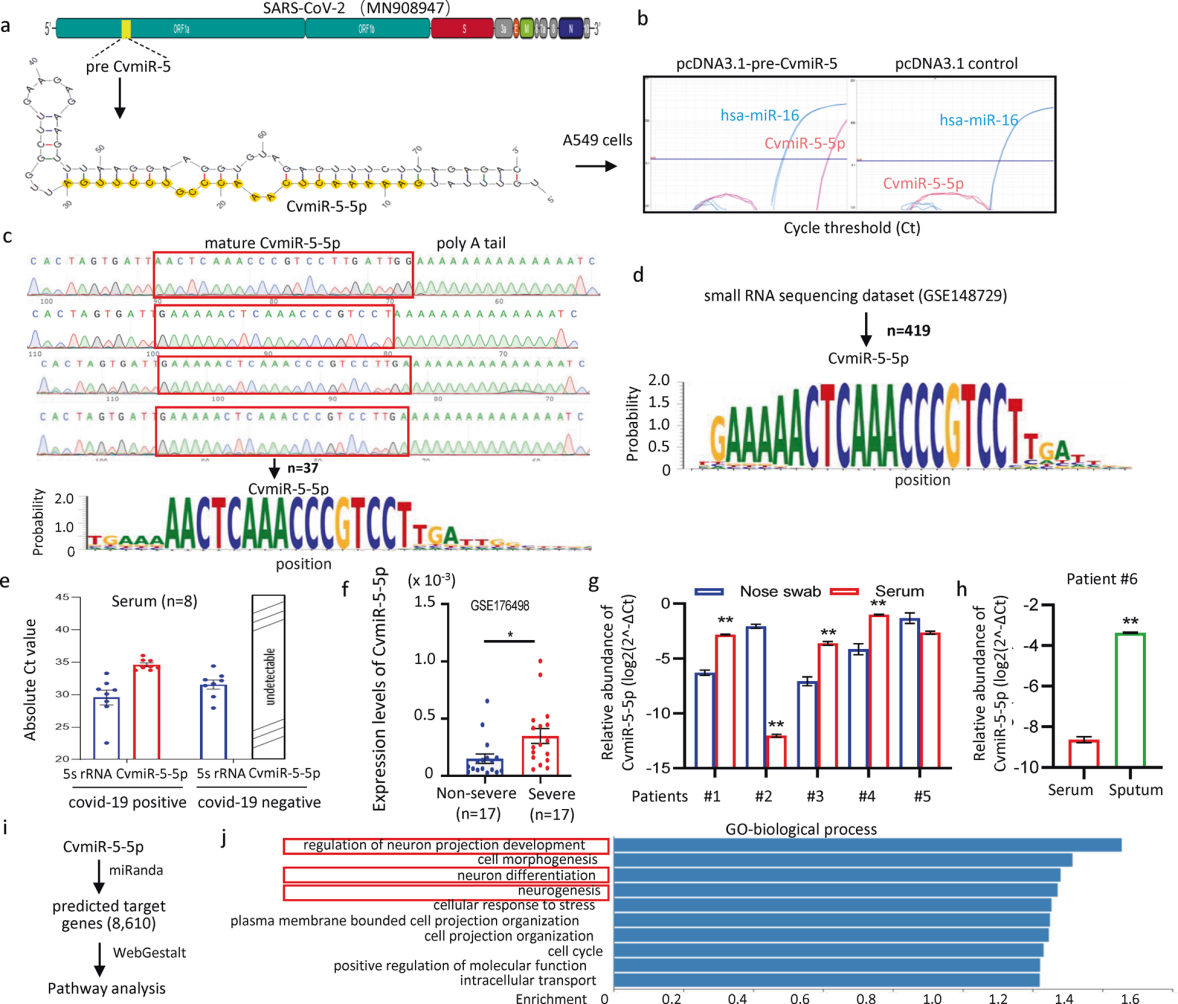
Identification of a novel vmiRNA encoded by SARS-CoV-2 genome in covid-19 patients. **a** Sequence location and the secondary structure of pre-CvmiR-5. **b** QRT-PCR analysis of CvmiR-5-5p in A549 cells transfected with pre-CvmiR-5 or vector control. Hsa-miR-16-5p served as an internal control for small RNAs. The amplification curves were indicated. **c** Sequencing validation of the PCR products of CvmiR-5-5p in **b**. The sanger sequencing graph was derived from 37 clones of CvmiR-5-5p amplified by PCR. **d** Sequence enrichment for CvmiR-5-5p motif derived from 419 reads in a small RNA sequencing dataset GSE148729 by analyzing the SARS-CoV-2 virus-infected Calu-3 cells. **e** Circulating CvmiR-5-5p analysis in eight serum samples from covid-19 patients (Patients #1–8) and eight serum samples from normal controls showing detectable in the patients with cycle threshold (Ct) values ~34–36, while undetectable in controls. 5s rRNA was used as an internal control of human small RNAs in circulation. **f** Comparison of the CvmiR-5-5p levels between non-severe patients and severe patients (*n* = 17 in each group). Data were derived from a public dataset GSE176498. **g** CvmiR-5-5p analysis in serum and nasal swab samples from five covid-19 patients (Patients #1–5) showing higher levels of CvmiR-5-5p in serums than nasal swabs in three of the five patients, while opposite trend in one of the five patients. **h** CvmiR-5-5p analysis in one pair sample of serum and sputum from covid-19 patient #6 showing a higher level of CvmiR-5-5p in the sputum than that in serum. **i** Schematic representation for target gene analysis of CvmiR-5-5p in human cells. **j** Pathways analysis by GO-biological process on the 8610 predicted potential target genes of CvmiR-5-5p indicating the main regulation of human nervous system including neurogenesis, neuron development, and neuron differentiation. All the analyses were performed in triplicates and repeated at least three times independently. Data were presented as the mean ± SEM. *p < 0.05; **p < 0.01

Next, quantitative comparisons of CvmiR-5-5p between the serum, sputum, and nasal swab samples were performed to determine the detection sensitivity. Eight serum samples from different covid-19 patients and eight serum samples from normal controls were applied for the detection of circulating CvmiR-5-5p. As shown in Fig. 1e and Supplementary Fig. S6, CvmiR-5-5p was detectable in all the serum samples of patients with cycle threshold (Ct) values of ~32–35, while undetectable in the serum from normal controls. In contrast, 5 s ribosome RNA (5 s rRNA), as an internal control of human small non-coding RNAs in circulation, showed Ct values ~28–34 in both groups (Fig. 1e). The results demonstrated the specificity of the CvmiR-5-5p-based detection of SARS-CoV-2 in serum of patients. Comparison of the CvmiR-5-5p levels between patients having different symptoms suggested a positive correlation between CvmiR-5-5p abundance and disease progression and severity (Fig. 1f and Supplementary Fig. S7). In order to compare the abundance of CvmiR-5-5p in different types of samples, we collected both serum and nasal swab samples from five covid-19 patients and applied for vmiRNA analysis. As a result, CvmiR-5-5p was well amplified in both serum and nasal swab samples (Supplementary Fig. S8). However, serum showed significantly higher levels of CvmiR-5-5p than nasal swab in three of the five patients (Fig. 1g). In contrast, only one of the five patients showed a lower level of CvmiR-5-5p in serum than that in nasal swab (Fig. 1g). Notably, the former three were asymptomatic patients, and the latter one was the only symptomatic patient among the five, suggesting the potential of the serum CvmiR-5-5p for early detection of viral infection before symptoms, which definitely needs more clinical samples for validation. In addition, we by chance collected a pair of serum and sputum samples from one covid-19 patient. The quantitative analysis showed a significantly higher level of CvmiR-5-5p in the sputum than that in serum (Supplementary Fig. S9 and 1h), suggesting sputum may be one type of specimen enriched with CvmiR-5-5p for easy and fast detection. Undoubtedly, this has to be further confirmed using a larger scale of samples. Notably, the availability of sputum samples has to be taken into account before being applied for detection.

Finally, we explored the involvement of CvmiR-5-5p in the pathogenesis of covid-19 patients. A well-designed miRNA target prediction tool, miRanda was applied, followed by the Gene Ontology (GO) terms and Kyoto Encyclopedia of Genes and Genomes (KEGG) pathways analyses (Fig. 1i). As a result, a total of 8610 mRNAs were predicted as the potential targets of CvmiR-5-5p in human host cells. Surprisingly, human nervous system-related pathways including regulation of neurogenesis, neuron development, and neuron differentiation were identified as the top five most enriched pathways in the GO-biological process analysis, (Fig. 1j). KEGG suggested the regulation of autophagy and multiple signaling pathways by CvmiR-5-5p and its target genes (Supplementary Fig. S10a). Between the predicted target genes and 3501 DEGs (differentially expressed genes) from RNA-seq analysis of SARS-CoV-2-infected cells,^6^ ~1700 genes were overlapped (Supplementary Fig. S10b). GO and KEGG analysis demonstrated their involvement in multiple signaling pathways in response to virus infection, neurotransmitter secretion, and so on (Supplementary Fig. S10c, d). Further overlapping with 86 DEGs in a proteomic analysis of the SARS-CoV-2-infected iPS-derived AT2s^6^ identified 6 human genes including TNFRSF10D, RRP15, NY-ESO1, FRMD4B, APLP2, and EPB41L4B (Supplementary Fig. S10e). The proteomic analysis indicated downregulation of RRP15, ESO1, FRMD4B, and APLP2, while upregulation of TNFRSF10D and EPB41L4B after SARS-CoV-2 infection (Supplementary Fig. S10e). Most likely the two upregulated genes are not direct targets of CvmiR-5-5p, or vmiRNAs may have a novel mechanism to regulate target genes differing from that in mammals. Notably, the gene APLP2 and its homologous family member amyloid precursor protein (APP) have been well demonstrated to play important roles in regulating the human neuron systems, such as neuron cell growth and Alzheimer’s disease. FRMD4B has been reported to enrich the brain to regulate neurological diseases.

In addition, RNA-seq analysis was applied to A549 cells overexpressing CvmiR-5 precursors. A subset of DEGs was identified (Supplementary Fig. S11). The pathway analysis further demonstrated the involvement of CvmiR-5 in regulating the immune system, nervous/sensory system, viral infection disease, and metabolic process (Supplementary Fig. S12). These findings strongly suggested a function of CvmiR-5-5p in regulating the pathogenesis of COVID-19, especially the occurrence of neurological disorders in COVID-19 patients.

Since neurological disorders including dysfunction of smell and taste, impaired consciousness and severe headache have been considered as common symptoms in covid-19 patients,^7^ we speculated that SARS-CoV-2-derived CvmiR-5-5p may be one of the key factors taking responsibility for the neurological symptoms in covid-19 patients. Targeted inhibition of CvmiR-5-5p by anti-vmiRNA oligonucleotides (ASO) may hold strong potential in the treatment of neurological symptoms. In conclusion, the current study not only identified a novel vmiRNA encoded by SARS-CoV-2 with the potential to serve as a diagnostic biomarker in the serum, nasal swab, and even sputum samples, but also provided a therapeutic strategy to treat covid-19 patients against neurological symptoms.

## Supplementary information

Supplementary Figures

## Data Availability

The online version of this article contains supplementary material, which is available to authorized users.
