# Supplementary material for: Identification of a SARS-CoV-2 virus-encoded small non-coding RNA in association with the neurological disorders in COVID-19 patients
Source: Signal Transduct Target Ther. 2022 Mar 31;7:107. doi: 10.1038/s41392-022-00969-1 (PMC8967939; doi:10.1038/s41392-022-00969-1)
Supplement: Supplementary file 1 — Supplementary Figures [file 41392_2022_969_MOESM1_ESM.docx]

Supplementary Materials for

Identification of a SARS-CoV-2 virus-encoded small non-coding RNA in association with the neurological disorders in COVID-19 patients

Qian Zhao1#, Qiong Wang1#, Bing Zhao2#, Yixing Wang1,3#, Jinhui Lü1, Yuefan Guo1, Xiaoping Zhu1, Lu Qian1, Shanzhan Yu1, Lipeng Hao2*, Zhongmin Liu1*, Zuoren Yu1*

^1^Key Laboratory of Arrhythmias of the Ministry of Education, Research Center for Translational Medicine, Heart Failure Institute, Shanghai East Hospital, Tongji University School of Medicine, Shanghai, China.

^2^Shanghai Pudong Center for Disease Control & Prevention, Pudong New Area, Shanghai, China.

^3^Department of Internal Medicine of Traditional Chinese Medicine, Tongji University School of Medicine, Shanghai East Hospital, Shanghai, China.

#Equal contribution

*Correspondence to: [zuoren.yu@tongji.edu.cn](mailto:zuoren.yu@tongji.edu.cn), [liu.zhongmin@tongji.edu.cn](mailto:liu.zhongmin@tongji.edu.cn), haolipeng_cc@163.com

**This PDF file includes:**

Materials and Methods

Supplementary Figures

Figures. S1 to S12

Materials and Methods

Sample collection

All the patients’ samples were collected by Shanghai Pudong Center for Disease Control & Prevention from covid-19 patients diagnosed by quantitative RT-PCR method. The negative control samples were collected by Shanghai East Hospital. The study was approved by the Institutional Review Board (IRB) of Shanghai East Hospital.

Cells and vectors

Double-stranded DNA of pre-CvmiR-5 was synthesized by Genescript (Genescript, Nanjing, China), and cloned into pcDNA3.1. Sequence of pre-CvmiR-5: 5’ UGUUUAUGAAAAACUCAAACCCGUCCUUGAUUGGCUUGAAGAGAAGUUUAAGGAAGGUGUAGAGUUUCUUAGAGAC 3’. The pseudovirus fragment starting from 5’ end to nt 2400 of SARS-CoV-2（MN908947.3）covering pre-CvmiR-5 was synthesized by Genescript and cloned into pcDNA3.1. Empty vector was used as a negative control. A549 cells were originally purchased from ATCC, and maintained in our lab. Lipofectamine 2000 (Invitrogen) was used for cell transfection.

RNA extraction from serum

1mL of Trizol reagent (Invitrogen, USA) was applied to an aliquot of 200μL serum for total RNA extraction following the standard protocol, followed by a quality analysis using Agilent Bioanalyzer 2100. Glycogen was used to help concentrating RNA pellets.

RNA extraction from sputum or nasal swab samples. MagNA Pure 96 DNA and Viral NA Small Volume Kit (Roche, Mannheim, Germany) was used for RNA extraction from sputum or nasal swab samples of covid-19 patients according to the manufacturer’s instruction.

QRT-PCR analysis

For nucleic acid test of covid-19 patients, 2019 novel CoronaVirus (ORF1ab/N gene) Real Time PCR Kit（Duplex PCR (BioGerm, Shanghai, China) was used for quantitative analysis following the manufacturer’s instruction. For vmiRNA analysis, 100ng of total RNA was applied to prepare the first strand cDNA of small non-coding RNAs by the method of poly A addition and reverse transcription as previously described [1]. SYBR Green Master Mix (Applied Biosystem, USA) and QuantStudio™ 6 Flex Real-Time PCR System (Applied Biosystem, USA) were used for real-time PCR analysis. 5s rRNA and hsa-miR-16-5p were used as internal controls for normalization. Forward primer sequences for CvmiR-5-5p: 5′ AACTCAAACCCGTCCTTGA 3′; 5 s rRNA, 5′ AGTACTTGGATGGGAGACCG 3′; hsa-miR-16-5p: 5′ TAGCAGCACGTAAATATTGGCG 3′. All primer oligos were synthesized by GenScript (Nanjing, China).

CvmiRNA prediction

Genome sequence of SARS-CoV-2 virus was obtained from NCBI GenBank (MN908947.3). 9,367 mature vmiRNA fragments in 55 pre-CvmiRNAs were predicted using miRPara6.3 [2]. By applying parameter filtering (SVM probability >0.99), 20 mature vmiRNA fragments in 15 precursors were filtered out for further analysis. The secondary structure of the pre-CvmiRNAs were predicted by mfold (http://www.unafold.org/).

MiRNA target prediction and pathway analysis

Target gene prediction of CvmiR-5-5p was performed using software of miRanda. Pathway analysis was performed using web-based gene set analysis toolkit (WebGestalt).

Public sequencing dataset

The dataset (GSE148729) of small RNA sequencing for the SARS-CoV-2 virus-infected Calu-3 cells [3] was used to validate the sequence of CvmiR-5-5p. The sequence logo diagrams were generated using an online tool (http://www.bioinformatics.com.cn). A published dataset of RNA-seq and a proteomic profiling in the SARS-CoV-2-infected iPS-derived AT2s (iAT2s) [4] were used for the target gene analysis of CvmiR-5-5p.

Statistical analysis. Data are presented as mean ± SEM unless otherwise stated. P<0.05 was considered as statistical signiﬁcance, which was determined using standard two-tailed student’s t-test.

**References**

1. Ding X, Li Y, Lü J, Zhao Q, Guo Y, Lu Z, et al. piRNA-823 Is Involved in Cancer Stem Cell Regulation Through Altering DNA Methylation in Association With Luminal Breast Cancer. Front Cell Dev Biol. 2021 Mar 15;9:641052. doi: 10.3389/fcell.2021.641052. eCollection 2021.

2. Wu Y, Wei B, Liu H, Li T, Rayner S. MiRPara: a SVM-based software tool for prediction of most probable microRNA coding regions in genome scale sequences. BMC Bioinformatics. 2011 Apr 19;12:107. doi: 10.1186/1471-2105-12-107.

3. Wyler E, Mösbauer K, Franke V, Diag A, Gottula LT, Arsiè R, et al. Transcriptomic profiling of SARS-CoV-2 infected human cell lines identifies HSP90 as target for COVID-19 therapy. iScience. 2021 Mar 19;24(3):102151. doi: 10.1016/j.isci.2021.102151. Epub 2021 Feb 6.

4. Hekman RM, Hume AJ, Goel RK, Abo KM, Huang J, Blum BC, et al. Actionable Cytopathogenic Host Responses of Human Alveolar Type 2 Cells to SARS-CoV-2. Mol Cell. 2020 Dec 17;80(6):1104-1122.e9. doi: 10.1016/j.molcel.2020.11.028. Epub 2020 Nov 19.

Supplementary Figures

Figure. S1.





**Figure S1:** Sequence blast analysis of eight kinds of coronavirus including SARS_CoV, MERS_CoV, HCoV_HKU1, HCoV_OC43, HCoV_NL63, HCoV_229E, SARS-CoV-2_Delta and SARS-CoV-2_Omicron versus SARS-CoV-2 indicated that CvmiR-5-5p sequence was specific to SARS-CoV-2, and conserved among the mutants of SARS-CoV-2.

Figure. S2.


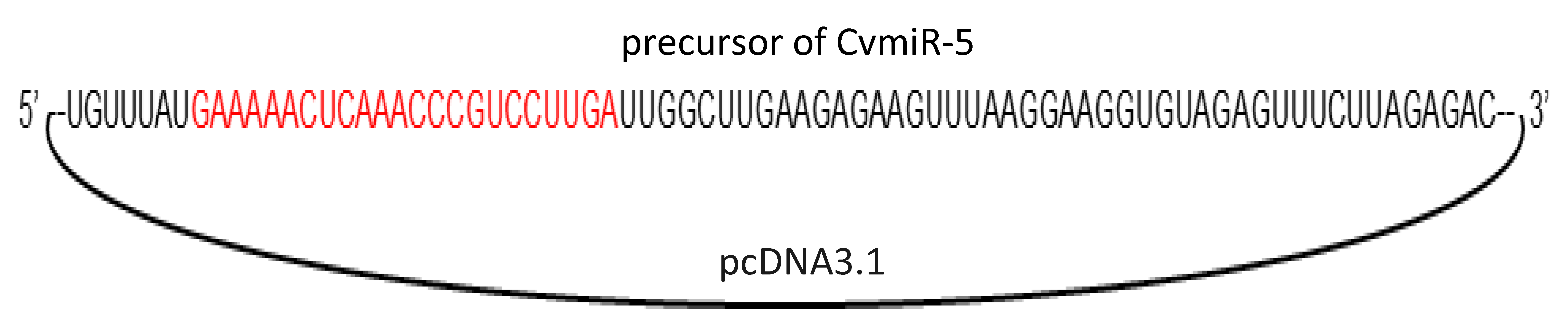


**Figure S2:** The RNA sequence of pre-CvmiR-5 and structure of the pcDNA 3.1 plasmid carrying pre-CvmiR-5. The sequence of predicted mature CvmiR-5-5p was highlighted in red.

Figure. S3.


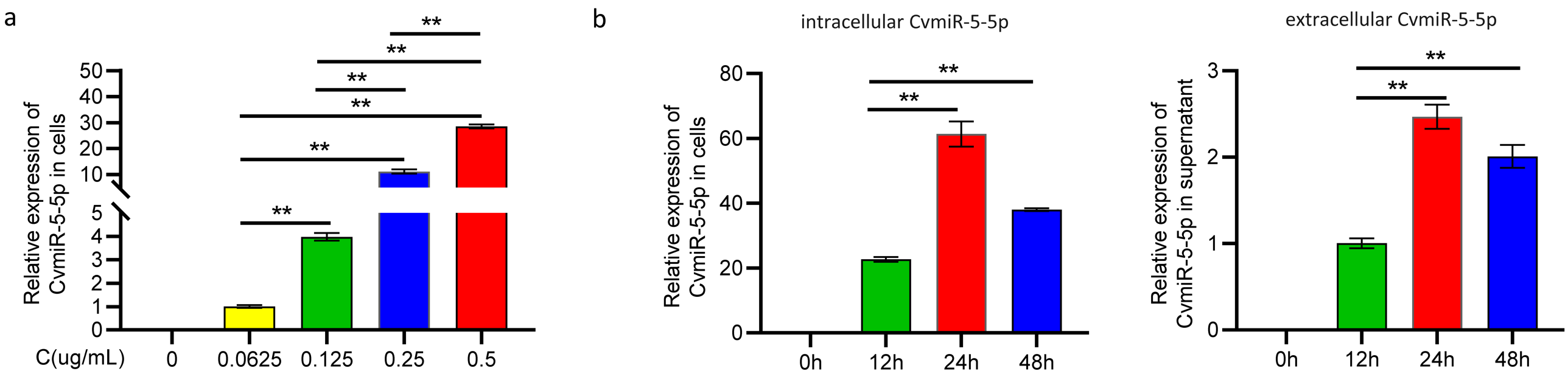
**Figure S3:** a. Quantitative analysis of CvmiR-5-5p in A549 cells in 24h after transfection with different concentration of pre-CvmiR-5. b. Quantitative analysis of intracellular and extracellular CvmiR-5-5p in A549 cells at different time points after transfection with 0.5 µg/ml of pre-CvmiR-5. CvmiR-5-5p in the supernatant showed similar pattern with that in the cells. Data are presented as the mean ± SEM (n=3). **p<0.01.

Figure. S4.


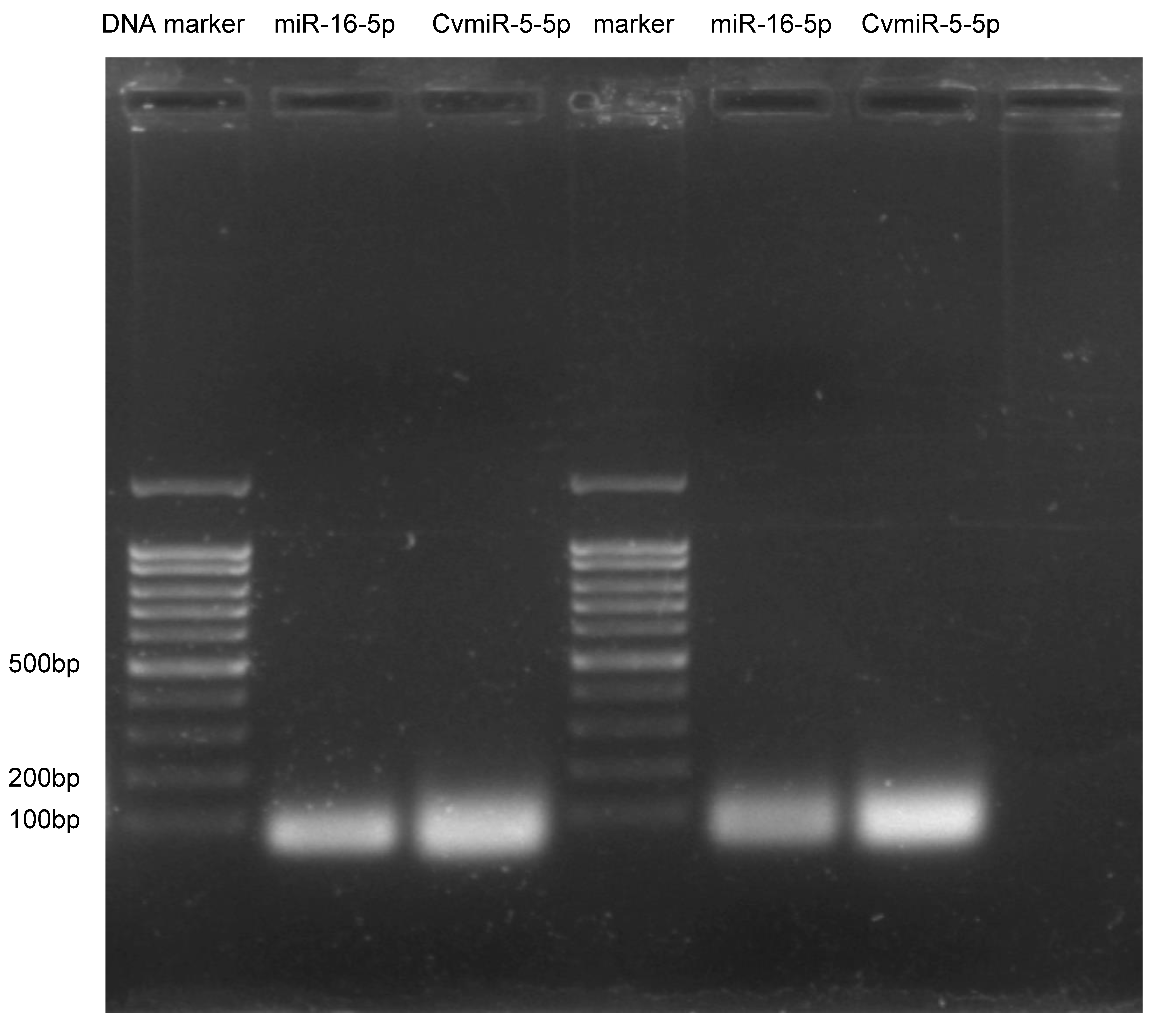


**Figure S4:** Agarose gel electrophoresis indicating the similar size between mature CvmiR-5-5p and internal control hsa-miR-16-5p of the PCR products.

Figure. S5.


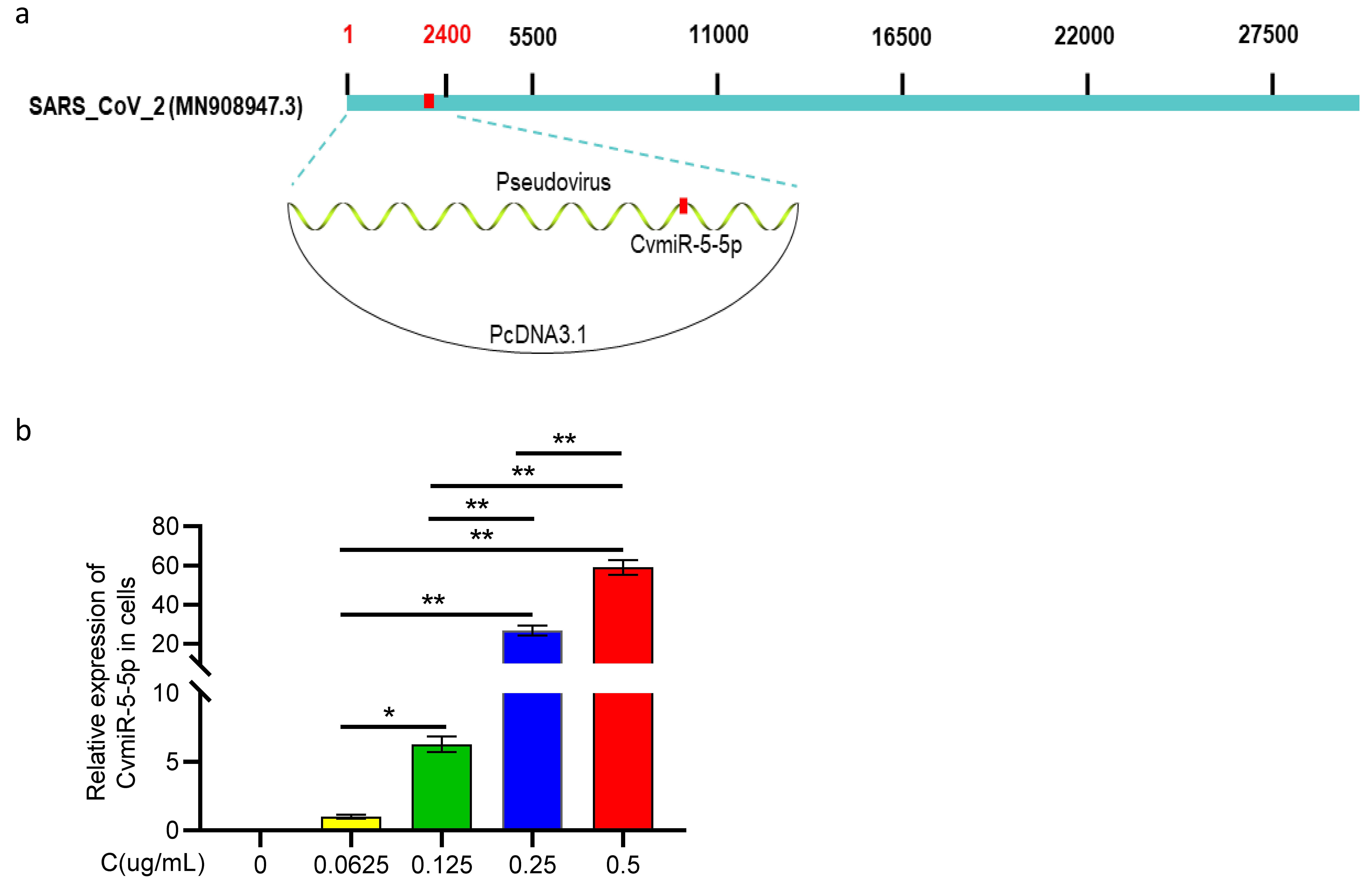
**Figure S5:** a. Schematic diagram of a pseudovirus fragment starting from 5’ end to nt 2400 of SARS-CoV-2 covering pre-CvmiR-5. b. Quantitative analysis of CvmiR-5-5p in A549 cells in 24h after transfection with different concentration of the pseudovirus. Data are presented as the mean ± SEM (n=3). * p<0.05; **p<0.01.

Figure. S6.





**Figure S6:** Amplification curves of CvmiR-5-5p by QRT-PCR analysis in 8 serum samples from covid-19 patients (Patients 1-8) (a) and 8 serum samples from normal controls (b). 5s rRNA served as internal control. Figure. S4.

Figure. S7.


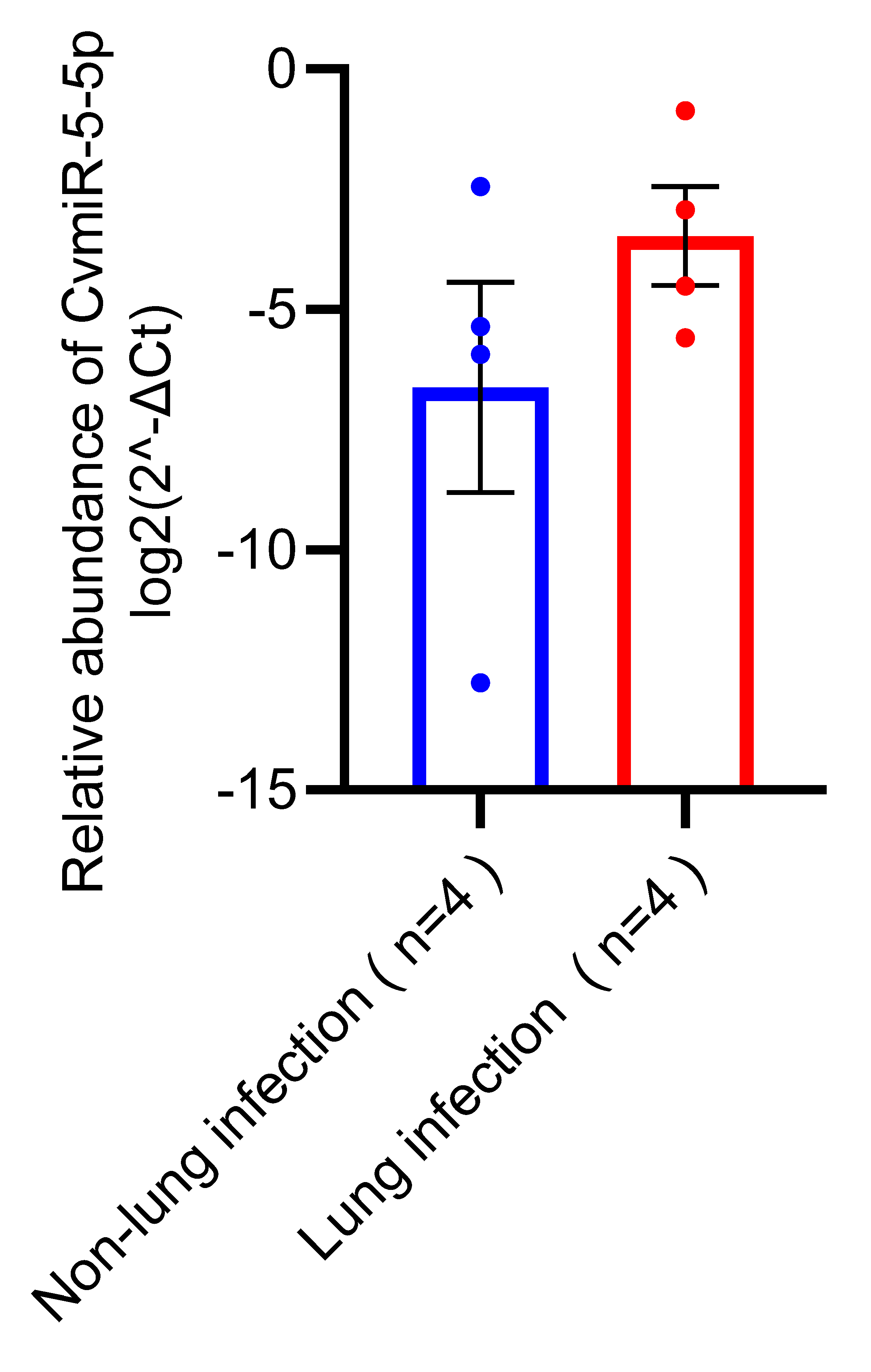


**Figure S7:** Comparison of the CvmiR-5-5p levels between the patients with (patients #1, #2, #4, #5) or without (patients #3, #6, #7, #8) infection in the lung. Along with the disease progression and infection in the lung, an increase trend of CvmiR-5-5p level was observed. More samples are required for statistical analysis.

Figure. S8.


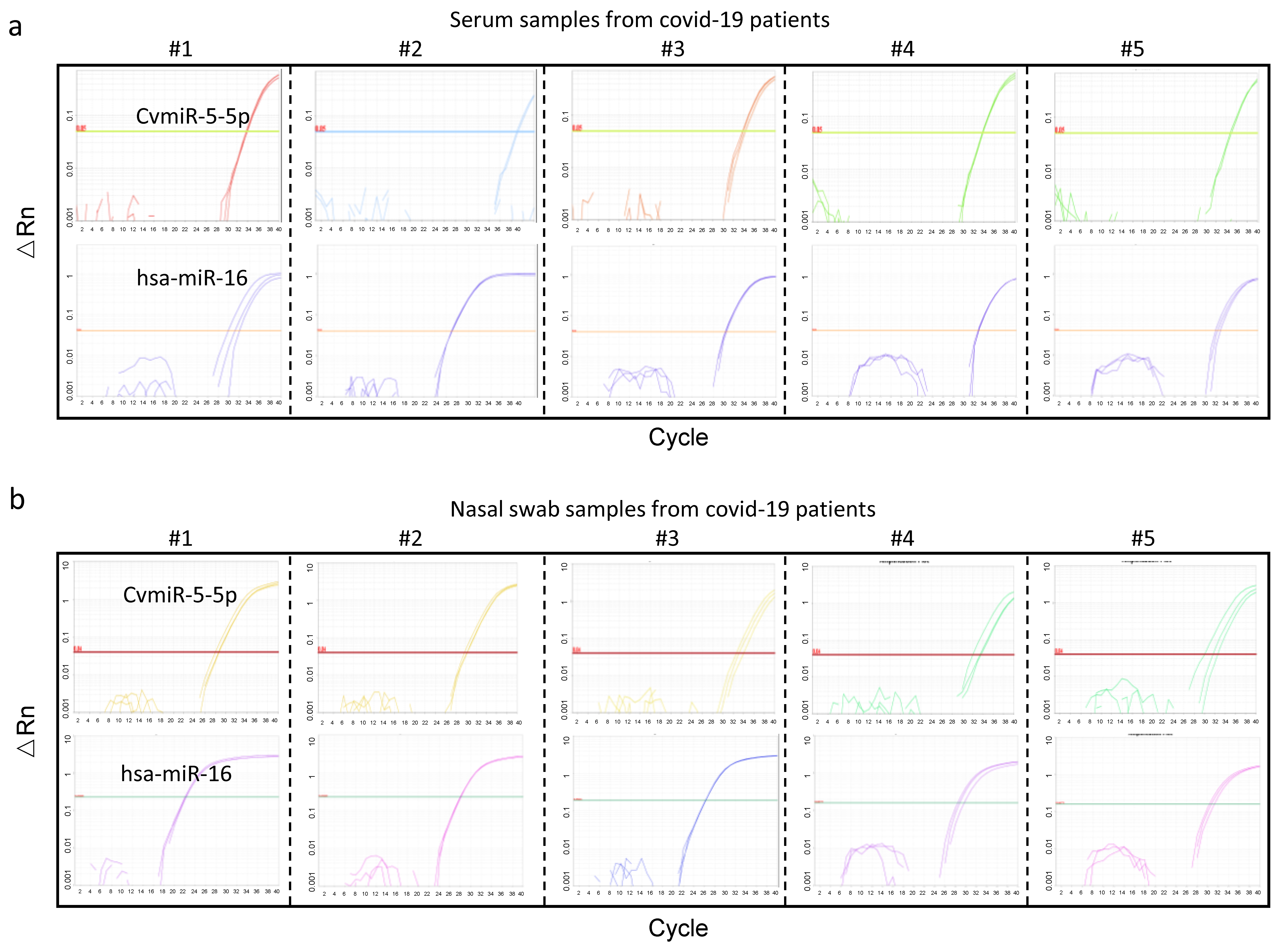
**Figure S8:** Amplification curves of CvmiR-5-5p by QRT-PCR analysis in the samples of serum (a) and nasal swab (b) from 5 covid-19 patients (Patients 1-5). Hsa-miR-16-5p served as internal control.

Figure. S9.


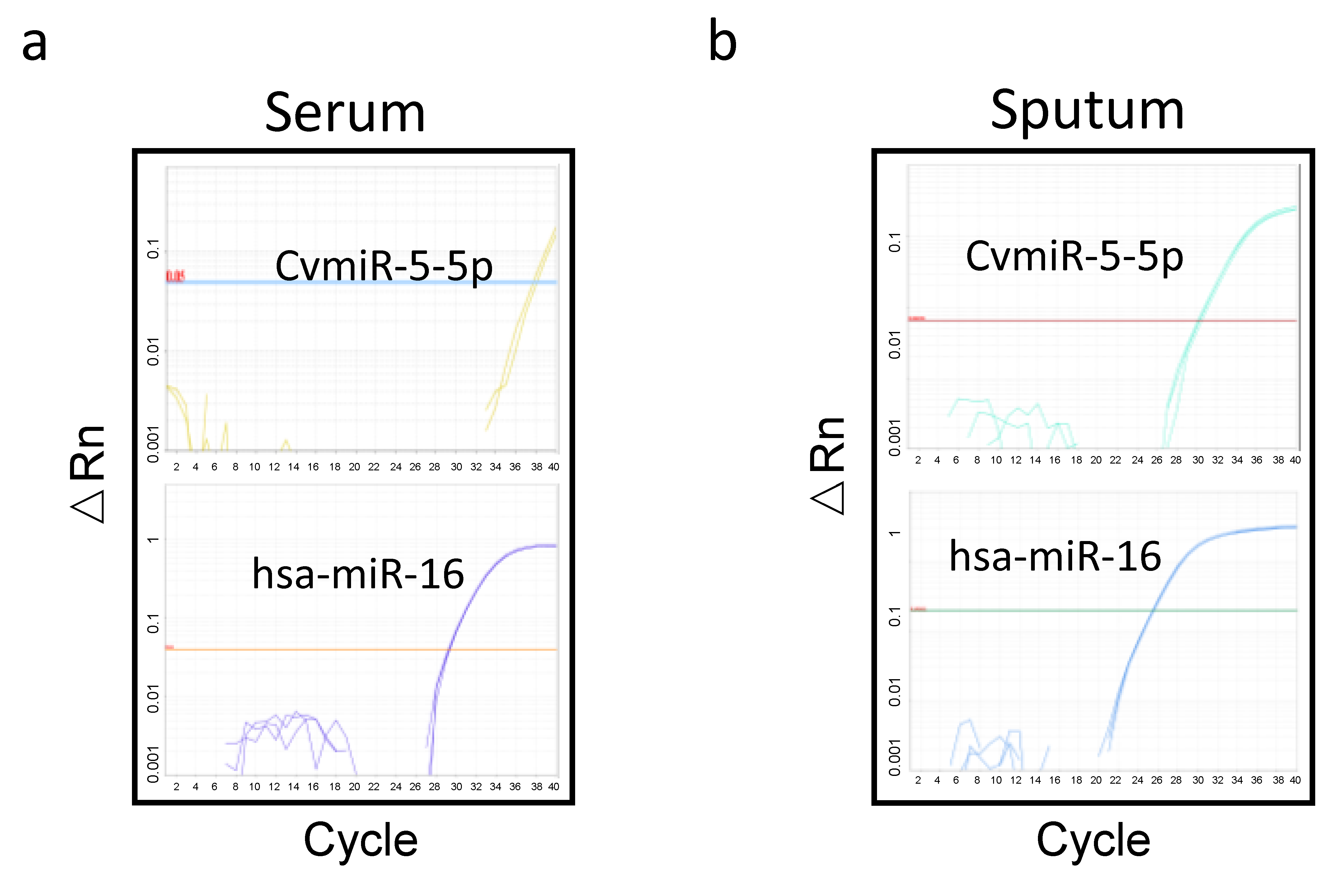


**Figure S9:** Amplification curves of CvmiR-5-5p by QRT-PCR analysis in the samples of serum (a) and sputum (b) from one covid-19 patient (Patient 6). Hsa-miR-16-5p served as internal control.

Figure. S10.



**Figure S10:** a. Pathway analysis of the predicted target genes of CvmiR-5-5p by KEGG. b. Six human genes including TNFRSF10D, FRMD4B, ESCO1, RRP15, APLP2 and EPB41L4B were overlapped among the 8,610 predicted potential target genes of CvmiR-5-5p, 3,501 DEGs in an RNA-seq analysis and 86 DEGs in a proteomic analysis of the SARS-CoV-2-infected human cells. C and D. KEGG (c) and GO (d) analyses of the 1,698 genes overlapped between the predicted target genes and 3,501 DEGs in the SARS-CoV-2-infected cells. e. Downregulation of ESCO1, RRP15, APLP2 and EPB41L4B, and upregulation of TNFRSF10D, FRMD4B at the protein levels in human cells by SARS-CoV-2 infection. Data are presented as the mean ± SEM (n=3). * p<0.05.

Figure. S11.


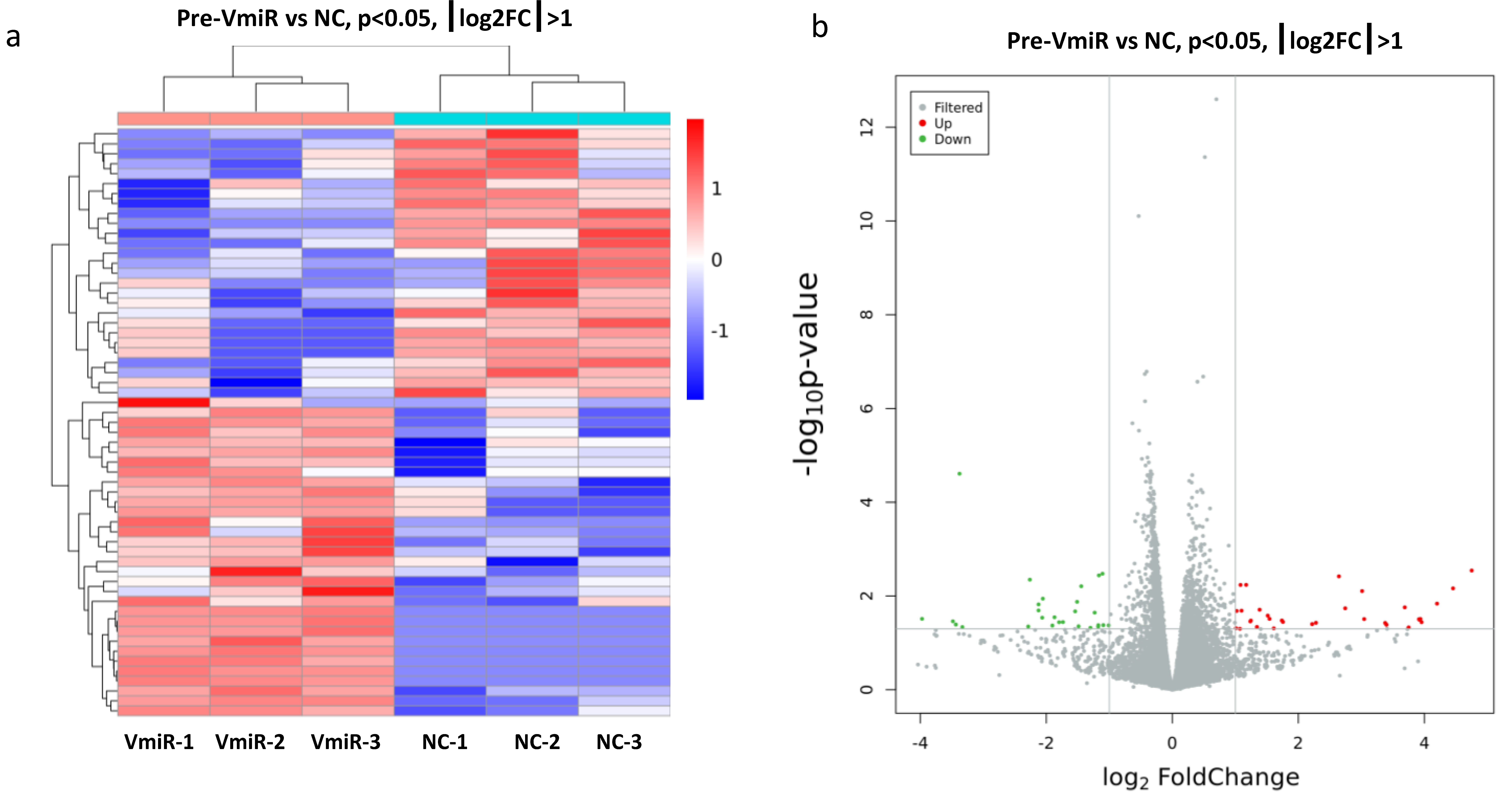
**Figure S11:** Heatmap (a) and Volcano plot (b) of a RNA-seq analysis to the A549 cells overexpressing CvmiR-5 precursor or control.

Figure. S12.



**Figure S12:** KEGG (a) and GO (b) analyses of the DEGs in Figure S11 indicated the enrichment in pathways regulating immune system, nervous/sensory system, viral infection disease, metabolic process, etc.
